# Lack of TAR-DNA binding protein-43 (TDP-43) pathology in human prion diseases

**DOI:** 10.1111/j.1365-2990.2008.00963.x

**Published:** 2008-08

**Authors:** A M Isaacs, C Powell, T E Webb, J M Linehan, J Collinge, S Brandner

**Affiliations:** * MRC Prion Unit, UCL Institute of NeurologyQueen Square, London, UK; † Department of Neurodegenerative Disease, UCL Institute of NeurologyQueen Square, London, UK; ‡ National Prion Clinic, National Hospital for Neurology and NeurosurgeryQueen Square, London, UK

**Keywords:** amyloid plaque, Creutzfeldt–Jakob disease, prion, TAR-DNA binding protein-43, ubiquitin, vCreutzfeldt–Jakob disease

## Abstract

**Aims:**

TAR-DNA binding protein-43 (TDP-43) is the major ubiquitinated protein in the aggregates in frontotemporal dementia with ubiquitin-positive, tau-negative inclusions and motor neurone disease. Abnormal TDP-43 immunoreactivity has also been described in Alzheimer's disease, Lewy body diseases and Guam parkinsonism–dementia complex. We therefore aimed to determine whether there is TDP-43 pathology in human prion diseases, which are characterised by variable deposition of prion protein (PrP) aggregates in the brain as amyloid plaques or more diffuse deposits.

**Material and methods:**

TDP-43, ubiquitin and PrP were analysed by immunohistochemistry and double-labelling immunofluorescence, in sporadic, acquired and inherited forms of human prion disease.

**Results:**

Most PrP plaques contained ubiquitin, while synaptic PrP deposits were not associated with ubiquitin. No abnormal TDP-43 inclusions were identified in any type of prion disease case, and TDP-43 did not co-localize with ubiquitin-positive PrP plaques or with diffuse PrP aggregates.

**Conclusions:**

These data do not support a role for TDP-43 in prion disease pathogenesis and argue that TDP-43 inclusions define a distinct group of neurodegenerative disorders.

## Introduction

TAR-DNA binding protein-43 (TDP-43) has recently been identified as the major protein in the ubiquitinated inclusions that characterize frontotemporal lobar degeneration with ubiquitin-positive, tau-negative inclusions (FTLD-U) and motor neurone disease (MND) [1]. TDP-43 pathology has since been reported in ubiquitinated inclusions in Alzheimer's disease cases [2,3], in Guam parkinsonism–dementia complex brains [4,5] and in Lewy body-related diseases [3,6]. Occasional TDP-43 staining has also been noted in neurofibrillary tangles [2,3,7] and in corticobasal degeneration [7] and Pick's disease brains [7,8]. These findings show that a number of neurodegenerative diseases characterized by protein aggregation have pathological TDP-43 immunoreactivity, prompting us to look in prion disease brains, which also contain protein aggregates.

Human prion diseases are a clinically and neuropathologically diverse group of neurodegenerative disorders in which conversion of host-encoded prion protein, termed PrP^C^, to an abnormally folded and aggregated form, termed PrP^Sc^, is a central feature [9,10].

Approximately 85% of human prion diseases occur as sporadic Creutzfeldt–Jakob disease (CJD) [11], and approximately 15% of human prion diseases are inherited, caused by mutations in the prion protein gene (*PRNP*) [9]. Susceptibility to sporadic prion diseases, as well as the acquired prion diseases described below, is affected by the genotype at codon 129 of *PRNP* [12–17]. Traditionally, the inherited cases have been classified as Gerstmann–Sträussler–Scheinker disease (GSS), fatal familial insomnia (FFI) or CJD; however, the range of clinical presentations, even within families with the same *PRNP* mutation [18–22], has led to sub-classification based on the *PRNP* mutation and the genotype at codon 129 [20,23].

Acquired prion diseases are a rare cause of prion disease in most populations, but can have a high frequency in certain populations. Acquired prion diseases can be classified as kuru, iatrogenic CJD or variant CJD (vCJD). Iatrogenic CJD has been caused by prion exposure from medical and surgical procedures, such as dura mater grafts, human growth hormone treatment and contamination of surgical equipment [24,25]. vCJD was first identified in the UK in 1995 as a novel variant of CJD [26], and generally has a much earlier age of onset than sporadic CJD [27]. The prion strain identified in vCJD has the same characteristics as bovine spongiform encephalopathy (BSE) prions, suggesting that dietary exposure to BSE-infected cattle was the cause for the vCJD outbreak [28–31]. There is now strong evidence that vCJD can also be transmitted by blood transfusion [32–34].

There are several characteristic neuropathological changes in the prion diseases described above: neuronal loss, astrocytic gliosis and spongiform vacuolation [35]. The latter can be absent in FFI [36] and can also be absent in severely ‘burnt-out’ areas, in which few neurons remain. Abnormal accumulation of PrP is generally observed, but the extent and form are variable [35] and, like spongiosis, is occasionally not observed in FFI [35,36]. GSS and kuru are characterized by PrP-positive amyloid plaques [35,37], while in sporadic CJD diffuse synaptic accumulation of PrP, which can also be seen in GSS, is more common [35,37]. vCJD is characterized by abundant florid amyloid plaques, in which the surrounding tissue is microvacuolated [26,38], but diffuse deposits can be seen as well.

Ubiquitin immunohistochemistry (IHC) in sporadic CJD and GSS has previously revealed staining at the periphery of amyloid plaques and around areas of spongiform change [39]. Because TDP-43 pathology can be associated with ubiquitinated deposits, and has now been observed in a number of neurodegenerative diseases, we analysed ubiquitin, PrP and TDP-43 immunoreactivity in different forms of prion diseases, including sporadic, inherited and acquired cases. PrP plaques were often accompanied by punctate ubiquitin deposits, but both PrP and ubiquitin staining were not associated with any TDP-43 cytoplasmic accumulation, suggesting that TDP-43 is probably not involved in prion disease pathogenesis.

## Materials and methods

### Use of human tissues

Human tissues were obtained at autopsy with consent for use in research. This study was approved by the UCL Institute of Neurology and National Hospital for Neurology and Neurosurgery Local Research Ethics Committee.

### Antibodies

Anti-TDP-43 (ProteinTech Group, Chicago, IL, USA), 1:10 000 for IHC, 1:150 for immunofluorescence (IF). Anti-ubiquitin (SC-8017, Santa Cruz Biotechnology, Santa Cruz, CA, USA) 1:5000 for IHC, 1:2500 for IF. Anti-PrP ICSM35 (D-Gen, London, UK) was raised in *Prnp*^o/o^ mice against recombinant β-PrP as previously described [40] and was used at 1:1000 for IHC and 1:500 for IF.

### Immunohistochemistry

Formalin-fixed brain samples were immersed in 98% formic acid for 1 h to denature infectious prions. Brain samples were then processed and 10-µm paraffin sections were cut and allowed to dry overnight at room temperature, then baked for 2 h at 60°C. Sections were pre-treated by boiling in 1 M citrate buffer pH 6.0 (TDP-43 and ubiquitin antibodies) and tris-EDTA-citrate buffer pH 7.8 (ICSM35). For specific detection of aggregated disease-associated PrP with ICSM35, sections were immersed in 98% formic acid for 5 min and treated with proteases to abolish the PrP^C^ signal. IHC was carried out on an automated immunostaining machine (Benchmark, Ventana Medical Systems Inc., Tucson, AZ, USA) using proprietary protease and secondary detection reagents and developed with 3′3 diaminobenzedine tetrachloride as the chromogen. Sections were counterstained with haematoxylin. Bright field photographs were taken on an ImageView II 3.5 Mpix digital camera (Soft Imaging Solutions GmbH, Münster, Germany, http://www.soft-imaging.de) mounted on a ZEISS Axioplan microscope (Carl Zeiss Jena GmbH, Jena, Germany) and composed with Adobe Photoshop (Adobe Systems Incorporated, San Jose, CA, USA).

### Double-labelling IF

Sections were pre-treated by pressure cooking for 20 min in 1 M citrate buffer pH 6.0 (ubiquitin and TDP-43 double labelling); 20-min pressure cooking in 1 M citrate buffer pH 6.0 followed by protease treatment (ubiquitin and ICSM35 double labelling); or pressure cooking for 20 min in 1 M citrate buffer pH 6.0 followed by 98% formic acid for 5 min and then protease treatment (TDP-43 and ICSM35 double labelling). Sections were manually stained sequentially for each primary antibody followed by detection with the relevant secondary antibodies; Alexa Fluor anti-rabbit 488; Alexa Fluor anti-mouse 546, Alexa Fluor anti-IgG1 488; Alexa Fluor anti-IgG2b 546 (all from Invitrogen, Carlsbad, CA, USA and used at 1:400). Laser scanning confocal microscopy was performed with a Zeiss LSM510 META, mounted on Zeiss Axiovert 200 M. All images were recorded using a ZEISS Plan-Apochromat 20x/0.75 objective. Image analysis was performed using Zeiss LSM software.

## Results

### PrP plaques but not diffuse PrP aggregates contain ubiquitin deposits

We initially examined PrP and ubiquitin immunoreactivity in the frontal cortex of a variety of inherited prion disease cases as well as sporadic, iatrogenic and variant CJD samples. In addition, we also analysed the cerebellum of a case with diagnosed GSS carrying a P102L mutation. Details of the cases examined are given in Table 1.

**Table 1:** Clinical and genetic data, and PrP and ubiquitin staining in the prion disease cases

Case No.	Figure	Prion disease type	Sex	Age at death (years)	Brain weight (g)	PRNP codon 129 genotype	PrP plaques	Synaptic PrP staining	Ubiquitin pathology
1	2 ABC	sCJD	M	67	1500	VV	0	+	None
2	3 GHI	sCJD	M	86	1445	MM	+	+++	Plaque associated
3	3 JKL	sCJD	F	59	1250	MM	0	+++	None
4	1 ABC	vCJD	F	56	1180	MM	++	0	Plaque associated
5	1 DEF 3 ABC	vCJD	M	18	1360	MM	+++	0	Plaque associated
6		vCJD	M	30	UA	MM	++	0	Plaque associated
7	1 GHI	iCJD	M	37	1405	MV	++*	++*	Plaque associated
8		Inherited prion disease – 6OPRI	F	49	UA	MV	0†	0†	None
9	2 JKL 3 MNO	Inherited prion disease – 6OPRI	M	43	1345	MV	+++	+++	Plaque associated
10	2 DEF	Inherited prion disease – A117V	F	46	1040	MM	0	++	None
11	2 MNO 3 DEF	Inherited prion disease – D178N (FFI)	M	60	1292	MM	++‡	0	Occasional plaque staining
12		Inherited prion disease – P102L (GSS)	M	64	1400	MM	+	++	Plaque associated
12	2 GHI 3 PQR	P102L (GSS) cerebellum					++	0	Plaque associated
13		Progranulin positive FTLD	F	66	UA	ND	ND	ND	Neurites and inclusions
14	1 JK	Progranulin positive FTLD	F	68	UA	ND	ND	ND	Neurites and inclusions

* Localized to deep cortical layers only.

† Axonal and dendritic PrP only.

‡ Diffuse PrP plaques.

Scoring for abnormal PrP immunoreactivity was as follows: 0, no staining; +, mild pathology; ++, moderate pathology, +++, severe pathology. Frontal cortex and hippocampus were analysed for all cases. Cerebellum was additionally examined in the P102L (GSS) case. All figures show frontal cortex staining except for the P102L (GSS) case for which cerebellum is shown. Column 1 indicates case number which is also shown in the figures and column 2 indicates the figures in which images of each case are shown.

FFI, fatal familial insomnia; FTLD, frontotemporal lobar degeneration; GSS, Gerstmann–Sträussler–Scheinker disease; iCJD, iatrogenic CJD from exposure to contaminated growth hormone; vCJD, variant CJD; sCJD, sporadic CJD; 6OPRI, six-octapeptide-repeat insertion – an insertion of 144 bp in the *PRNP* gene, coding for six repeats of an octapeptide motif [41]; UA, unavailable; ND, not determined.

**Figure 1:**
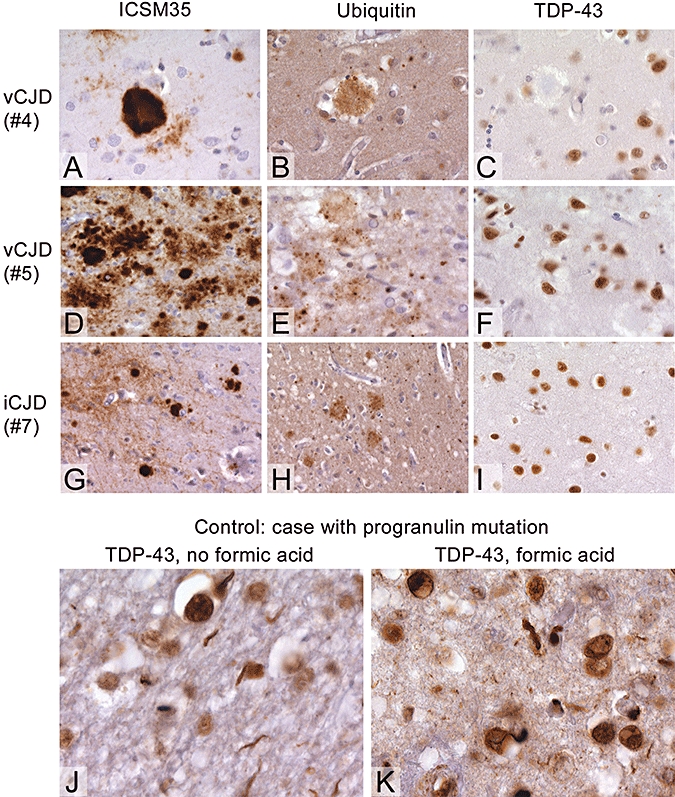
Prion protein (PrP), ubiquitin and TAR-DNA binding protein-43 (TDP-43) staining in acquired prion diseases: In all cases, prion protein plaques co-localize with ubiquitin aggregates, but no TDP-43 abnormalities are observed. (**A**–**C**) Variant Creutzfeldt–Jakob disease (vCJD) with characteristic dense PrP deposits surrounded by vacuolations (‘florid plaques’). Even in close proximity to the plaques, there is no abnormal nuclear deposition of TDP-43. (**D**–**F**) Abundant plaques in another vCJD case, again co-localizing with ubiquitin granular deposits. (**G**–**I**) Synaptic and plaque deposits in iatrogenic CJD, which contain ubiquitin but not TDP-43. Progranulin-positive frontotemporal lobar degeneration with ubiquitin-positive, tau-negative inclusions (FTLD-U) case showing neuritic pathology in the absence (**J**), or presence (**K**) of formic acid treatment. An intranuclear inclusion is also apparent in the formic acid-treated sample. Numbers indicate case numbers (see Table 1). Scale bar: 50 µm for images **A**–**F**, 100 µm for images **G** and **H** and 36 µm for **J** and **K**.

**Figure 2:**
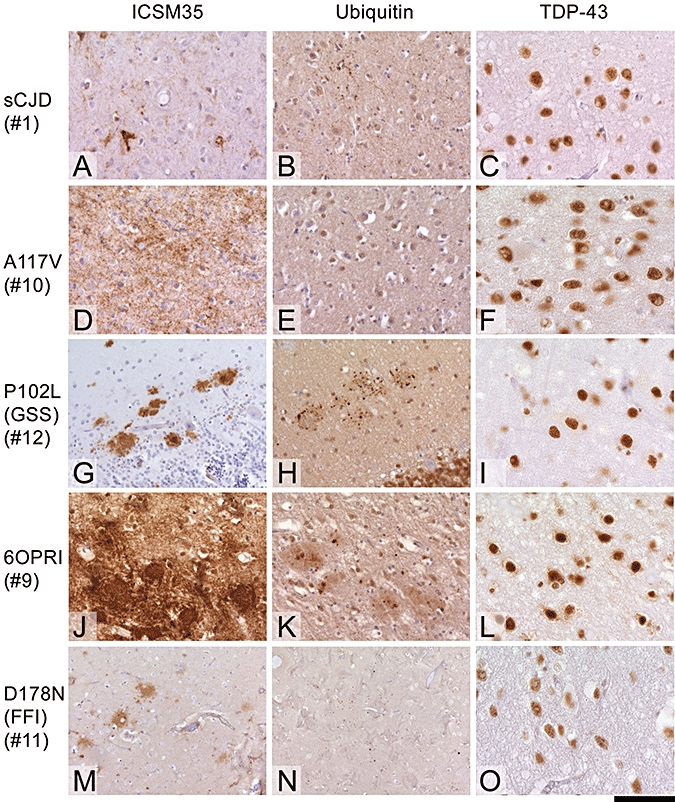
Prion protein (PrP), ubiquitin and TAR-DNA binding protein-43 (TDP-43) staining in sporadic and inherited prion diseases. (**A**–**C**) sCJD with synaptic PrP deposition, very little punctate ubiquitin deposits and normal TDP-43 labelling. (**D**,**G**,**J**,**M**) Inherited prion diseases with various types of prion protein deposits. (**D**–**F**) A117V mutation with diffuse, synaptic deposits, again with no marked ubiquitin deposition and no TDP-43 abnormalities. (**G**–**I)** GSS with P102L mutation shows marked ubiquitin aggregates in and around plaques. (**J**–**L**) Case of six-octapeptide-repeat insertion (144-bp insert) with abundant, dense prion protein deposits, mainly presenting with ‘primitive’ plaques (**J**), which are accompanied by ubiquitin aggregates (**K**), but no changes in TDP-43. (**M**–**O**) FFI with diffuse PrP plaques (**M**), no ubiquitin and no TDP-43 abnormalities. Numbers below the disease type indicate case numbers (see Table 1). Scale bar 100 µm for all images except **C**, **I**, **F**, **L** and **O** for which the scale bar is 50 µm.

**Figure 3:**
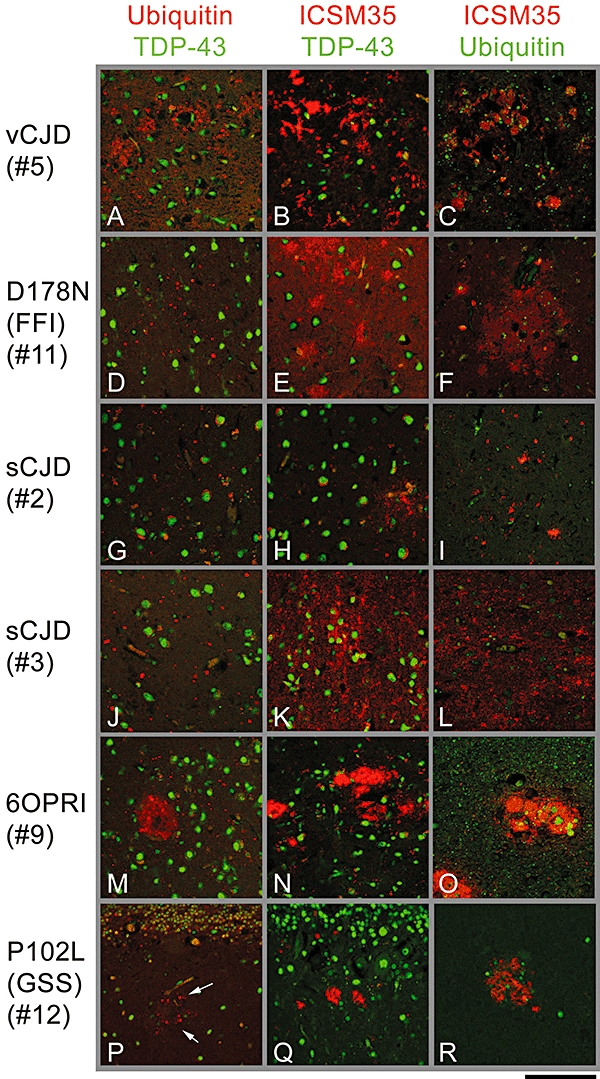
Double-labelling immunofluorescence for prion protein (PrP), ubiquitin and TAR-DNA binding protein-43 (TDP-43). Demonstration of close association of PrP and ubiquitin in plaques, but not in synaptic deposits. No TDP-43 abnormalities in cases of synaptic or plaque deposits. (**A**–**C**) Variant Creutzfeldt–Jakob disease with abundant florid plaques (see also Figure 1**D–F**) shows numerous granular ubiquitin deposits with no impact on nuclear labelling of TDP-43 (**A**), likewise no TDP-43 abnormality around PrP deposits (**B**). (**C**) Close association of ubiquitin with PrP labelling. The slightly weaker ubiquitin labelling than in (**A)** is a result of the different pre-treatment of the sections to visualize PrP, resulting in some quenching of ubiquitin immunoreactivity. (**D**–**F**) FFI with similar findings as in (**A–C**). The area void of TDP-43 labelling in the centre of the plaque is due to the loss or displacement of neurons in the area of the plaque. **E** and **F** as in **B** and **C**. **G**–**L** show little ubiquitin staining due to the absence of PrP plaques. In contrast, prion diseases with abundant plaques show more abundant ubiquitin in plaques, and a reduction of TDP-43 in the very centre of the plaque, due to neuronal loss (**M,P**). Arrows in **P** indicate punctate ubiquitin staining around a plaque. (**Q**) Gerstmann–Sträussler–Scheinker disease plaques in the cerebellar molecular layer (the granular layer is on top). (**O**,**R**) Close association of ubiquitin deposits with plaque protein. Scale bar, 100 µm for all images except **I** (200 µm).

Disease-associated PrP was detected with the monoclonal antibody ICSM35 as described in the methods. We scored the abundance of PrP plaques and diffuse PrP aggregates for each case in a semi-quantitative assessment, and analysed ubiquitin pathology in these scored cases. As expected, vCJD brains contained abundant PrP-positive plaques (Figure 1**A**,**D**), as did one inherited prion disease case with a six-octapeptide-repeat insertion (6OPRI) in *PRNP* (Figure 2**J**). Interestingly, another case with the same 6OPRI insertion mutation had no PrP plaques, but rather fine axonal and dendritic PrP staining in grey and white matter, confirming the neuropathological heterogeneity even of cases with the same mutation (data not shown). The P102L (GSS) brain contained mature PrP plaques (Figure 2**G**), while the D178N (FFI) case had more diffuse or primitive plaques (but no core plaques) (Figure 2**M**). PrP plaques were consistently dotted with small ubiquitin aggregates, except in the D178N (FFI) case in which the correlation between the diffuse plaques and ubiquitin deposits was less obvious (Figure 2**N**). The plaques could be clearly identified by ubiquitin immunoreactivity using both standard IHC and IF (Figure 3). Double labelling confirmed the presence of small ubiquitin deposits within PrP plaques (Figure 3), and also showed that some D178N (FFI) plaques contained ubiquitin deposits (Figure 3**F**).

Diffuse synaptic PrP aggregates were observed in the sporadic CJD cases as well as in other cases, but did not show any apparent co-localization with ubiquitin (Table 1, Figures 1 and 2). The lack of co-localization between diffuse PrP aggregates and ubiquitin was confirmed by double-labelling IF (Figure 3). These data suggest that PrP plaques, but not diffuse PrP pathology, contain ubiquitin deposits.

### PrP and ubiquitin aggregates in human prion diseases are TDP-43-negative

The TDP-43 immunoreactivity was initially analysed in the frontal cortex of all of the prion disease cases described. TDP-43 was nuclear in all cases, and did not appear to co-localize with diffuse PrP, PrP plaques or plaque-associated ubiquitin deposits in any of the cases (Figures 1 and 2). No co-localization of TDP-43 with ubiquitin, or TDP-43 with PrP aggregates was seen using double-labelling IF (Figure 3).

Abnormal TDP-43 accumulation has also been reported in the hippocampus in Alzheimer's disease and dementia with Lewy bodies [3]. Therefore, we also analysed TDP-43 immunoreactivity in the hippocampus in all cases, and similarly to frontal cortex found no abnormalities (data not shown). TDP-43 pathology has been reported in the absence of ubiquitin immunoreactivity in both white and grey matter of FTLD-U cases [42–44] and Guam parkinsonism–dementia complex cases [5]. We examined TDP-43 in the white matter and in grey matter areas not associated with PrP pathology in both hippocampus and frontal cortex, but did not observe any abnormal TDP-43 staining. Re-localization of TDP-43 from the nucleus to cytoplasmic aggregates appears to be a characteristic finding in TDP-43 proteinopathies, but reduced nuclear staining due to granular cytoplasmic TDP-43 immunoreactivity, rather than distinct inclusions, has also been reported [42,43,45]. We therefore also examined the frontal cortex and hippocampus of prion disease cases for instances of cytoplasmic rather than nuclear TDP-43 localization, but did not observe any such staining.

One possible confounding factor is that all formalin-fixed prion disease brains were immersed in formic acid prior to processing to abolish prion infectivity. To exclude the possibility that formic acid treatment alters pathological TDP-43 immunoreactivity (normal nuclear staining of TDP-43 was very strong in formic acid-treated brains), we analysed two progranulin-positive FTLD-U cases [46]. For each FTLD-U case, two samples of frontal cortex were processed separately, either with or without formic acid treatment. Immunostaining revealed extensive TDP-43 pathology in both the formic acid-treated and untreated samples (Figure 1**J**,**K**), confirming that the lack of TDP-43 pathology in our prion disease brains was not due to formic acid treatment. These data therefore show that TDP-43 pathology is not observed in a wide range of prion brains with differing PrP pathology.

## Discussion

TDP-43 was initially shown to be the major ubiquitinated protein in the inclusions in FTLD-U and MND [1]. Subsequently, TDP-43 pathology was identified in Alzheimer's disease, Guam parkinsonism–dementia complex and Lewy body diseases [2–6]. It is therefore important to define the range of neurodegenerative diseases with protein aggregates that harbour TDP-43 pathology.

Prion disease brains usually contain PrP aggregates, and a previous study showed ubiquitin staining in the periphery of plaques in GSS and sporadic cases, as well as ubiquitin immunoreactivity around areas of spongiform change [39]. We have now extended the analysis of ubiquitin in prion disease brains by examining a wide range of human prion disease cases including variant CJD and a variety of inherited cases with distinct pathological features. We did not observe ubiquitin in areas of spongiform change, as was previously described, which may be due to differences in antibody specificity or sensitivity, or the particular staining and pre-treatment protocols used. We show here that PrP plaques, but not diffuse (synaptic) PrP aggregates, contain ubiquitin deposits. Interestingly, the more diffuse PrP plaques in FFI stained less often for ubiquitin than more compact PrP plaques, possibly indicating that compaction of plaques facilitates their ubiquitination or that ubiquitin is sequestered into compact plaques.

We examined a number of different cases of prion disease with ubiquitin-positive PrP plaques. Ubiquitin deposits were always negative for TDP-43, and TDP-43 did not show abnormal cytoplasmic aggregation nearby or distant from plaques. TDP-43 was also normal in areas with diffuse PrP aggregates and in areas without any abnormal PrP immunoreactivity, including the white matter. These data suggest that TDP-43 is probably not involved in the pathogenesis or progression of prion diseases. TDP-43 pathology, therefore, appears to be specific to a subset of neurodegenerative diseases, which may share some aetiological features. Understanding the role and significance of TDP-43 in these cases is now a fundamental question in the field, and helping to define the clinical spectrum of TDP-43 proteinopathies, as we have done here, is an important first step in this process. Prion diseases and TDP-43 proteinopathies share the feature of protein aggregation, and there is some evidence that *PRNP* may have a role in Alzheimer's disease [47–49]. There is also conflicting data on whether *PRNP* genotype can affect the risk of FTD [50–52]. However, the lack of TDP-43 pathology in prion diseases suggests that if there is a common pathogenic pathway with TDP-43 proteinopathies, it is downstream of TDP-43. This could also be the case with certain disorders that are in the clinical and neuropathological spectrum of TDP-43 proteinopathies but which are TDP-43-negative, such as SOD1-positive MND [53,54] and certain FTLD-U cases [42,55].

In conclusion, human prion diseases do not have detectable TDP-43 pathology, further defining the spectrum of TDP-43 proteinopathies.
